# Correction: MALDI-IMS combined with shotgun proteomics identify and localize new factors in male infertility

**DOI:** 10.26508/lsa.202101015

**Published:** 2021-01-21

**Authors:** Shibojyoti Lahiri, Wasim Aftab, Lena Walenta, Leena Strauss, Matti Poutanen, Artur Mayerhofer, Axel Imhof

**Affiliations:** 1 Biomedical Center, Protein Analysis Unit, Faculty of Medicine, Ludwig-Maximilians-Universität München, Planegg-Martinsried, Germany; 2 Biomedical Center, Cell Biology-Anatomy III, Ludwig-Maximilians-Universität München, Planegg-Martinsried, Germany; 3 Institute of Biomedicine, Research Centre for Integrative Physiology and Pharmacology and Turku Center for Disease Modeling, University of Turku, Turku, Finland; 4 Graduate School for Quantitative Biosciences (QBM), Ludwig-Maximilians-Universität Munich, Munich, Germany

## Abstract

Correction

See original article: MALDI-IMS combined with shotgun proteomics identify and localize new factors in male infertility, 4(3), 2021.

*LSA* regrets that in the original version of this article, the labels for mass were mistakenly listed as kilodaltons (kD) instead of dalton (Da). The errors appear only in PDFs downloaded before January 7, 2021.

## Supplementary Material

Reviewer comments

