# Supplementary material for: Correction: MALDI-IMS combined with shotgun proteomics identify and localize new factors in male infertility
Source: Life Sci Alliance. 2021 Jan 21;4(3):e202101015. doi: 10.26508/lsa.202101015 (PMC7918713; doi:10.26508/lsa.202101015)
Supplement: Reviewer comments [file LSA-2021-01015_review_history.pdf]

Life Science Alliance

Correction: MALDI-IMS combined with shotgun proteomics identify and localize new factors in male ...

Axel Imhof, Shibojyoti Lahiri, Wasim Aftab, Lena Walenta, Leena Strauss, Matti Poutanen, and Artur Mayerhofer
DOI: https://doi.org/10.26508/lsa.202101015

Corresponding author(s): Axel Imhof, Ludwig-Maximilians-University of Munich

Review Timeline:

Submission Date:	2021-01-08
Accepted:	2021-01-08

Scientific Editor: Shachi Bhatt

Transaction Report:

(Note: With the exception of the correction of typographical or spelling errors that could be a source of ambiguity, letters and reports are not edited. The original formatting of letters and referee reports may not be reflected in this compilation.)

January 8, 2021

RE: Life Science Alliance Manuscript #LSA-2021-01015

Dr. Axel Imhof
Ludwig-Maximilians-University of Munich
Munich Center of Integrated Protein Science and BioMedical Centre
Großhadernerstr. 9
Planegg-Martinsried 82152
Germany

Dear Dr. Imhof,

This is to inform you that we are issuing a Correction entitled "Correction: MALDI-IMS combined with shotgun proteomics identify and localize new factors in male ...". for your manuscript, to notify everyone labels for mass were mistakenly listed as kilodaltons (kD) instead of dalton (D).

Apologies for this inconvenience.

Sincerely,

Shachi Bhatt, Ph.D.
Executive Editor
Life Science Alliance
https://www.lsajournal.org/
Tweet @SciBhatt @LSAjournal

January 8, 2021

RE: Life Science Alliance Manuscript #LSA-2021-01015

Dr. Axel Imhof
Ludwig-Maximilians-University of Munich
Munich Center of Integrated Protein Science and BioMedical Centre
Großhadernerstr. 9
Planegg-Martinsried 82152
Germany

Dear Dr. Imhof,

This is to inform you that we are issuing a Correction entitled "Correction: MALDI-IMS combined with shotgun proteomics identify and localize new factors in male ...". for your manuscript, to notify everyone labels for mass were mistakenly listed as kilodaltons (kD) instead of dalton (D).

Apologies for this inconvenience.

Sincerely,

Shachi Bhatt, Ph.D.
Executive Editor
Life Science Alliance
https://www.lsajournal.org/
Tweet @SciBhatt @LSAjournal
